# Exploratory Associations of Inflammatory Cytokines, Brain-Derived Neurotrophic Factor, and Vascular Endothelial Growth Factor with Clinical Outcomes in Patients with Bipolar Disorder

**DOI:** 10.3390/metabo16050328

**Published:** 2026-05-14

**Authors:** Fumito Hamada, Leo Gotoh, Yuko Tomiyama, Hiroko Sugawara, Muneaki Ogata, Hiroki Kumagai, Ryo Asada, Ryusei Hatae, Kiyohiro Yasumatsu, Hikaru Hori

**Affiliations:** 1 Department of Psychiatry, Faculty of Medicine, Fukuoka University, 7-45-1 Nanakuma, Jyonan-ku, Fukuoka City 8140180, Fukuoka, Japan; hamada2310@adm.fukuoka-u.ac.jp (F.H.); tomiyama@adm.fukuoka-u.ac.jp (Y.T.); h.sugawara.se@adm.fukuoka-u.ac.jp (H.S.); mu1218ne@adm.fukuoka-u.ac.jp (M.O.); kumagaihiroki@adm.fukuoka-u.ac.jp (H.K.); r.asada.ul@adm.fukuoka-u.ac.jp (R.A.); r.hatae.bn@adm.fukuoka-u.ac.jp (R.H.); k.yasumatsu.ov@adm.fukuoka-u.ac.jp (K.Y.); 2 Department of Psychiatry, Shiranui Hospital, Tegama, Omuta City 8360004, Fukuoka, Japan; gleo@adm.fukuoka-u.ac.jp

**Keywords:** brain-derived neurotrophic factor, bipolar disorder, cytokines, vascular endothelial growth factor

## Abstract

**Highlights:**

**What are the main findings?**

- Subjective cognitive dysfunction was strongly associated with depressive symptoms and reduced quality of life in bipolar depression.
- Exploratory biomarker analyses suggested association of IL-β and quality of life remained significant after FDR correction.

**What are the implications of the main findings?**

- Subjective cognitive function may be an important therapeutic target in bipolar disorder.
- There is a need for research on biomarkers related to recovery from bipolar disorder and quality of life.

**Abstract:**

Background/Objectives: Bipolar disorder is characterized by psychosocial dysfunction, cognitive impairment, and incomplete recovery. Although inflammatory and neurotrophic mechanisms have been implicated, their relationships with multidimensional recovery outcomes remain unclear. We examined the relationships of inflammatory cytokines, brain-derived neurotrophic factor (BDNF), and vascular endothelial growth factor (VEGF) with depressive symptoms, psychosocial functioning, cognitive performance, personal recovery, and quality of life (QOL) in patients with bipolar disorder. Methods: This cross-sectional study of 24 patients with bipolar disorder assessed depressive symptoms, psychosocial functioning, cognitive functions, personal recovery, and QOL. Plasma tumor necrosis factor alpha, interleukin (IL)-6, IL-1β, IL-2, BDNF, and VEGF-A were measured by assay. Results: Subjective cognitive dysfunction was significantly associated with depressive symptom severity (rho = 0.53, *p* = 0.0083) and reduced QOL (rho = −0.56, *p* = 0.0042). Depressive symptoms were also associated with lower WHO-QOL-26 scores (rho = −0.43, *p* = 0.038). Significant interrelationships were observed among objective cognitive measures, and after false discovery rate (FDR) correction, the associations between FAST and PDQ-5-D, Symbol Check and Codebreaker, and Codebreaker and Trail remained statistically significant. High plasma IL-6 levels were associated with worse executive function (rho = 0.43, *p* = 0.0068). Higher VEGF levels were associated with better attentional performance (rho = −0.42, *p* = 0.042). Plasma IL-1β levels were positively associated with QOL (rho = 0.54, *p* = 0.02). After FDR correction, only the association between IL-1β and QOL remained statistically significant. Conclusions: This pilot study suggests that there may be associations between cognitive impairment and cytokines, as well as between quality of life and VEGF, in bipolar disorder. Further studies with larger sample sizes are needed.

## 1. Introduction

Bipolar disorder (BD) is a chronic episodic mood disorder associated with substantial psychosocial disability and reduced quality of life (QOL) [1,2,3,4]. It imposes high societal costs, with indirect costs, such as inability to work or sustain employment, exceeding direct costs [4,5,6]. Treatment for BD aims to alleviate mood symptoms, achieve functional and social recovery, and improve quality of life. Depression is the most prevalent and disabling symptom in this disorder, accounting for the majority of symptomatic time. It is also the major determinant of functional impairment, cognitive dysfunction, and incomplete recovery [5,6]. Even when depressive symptoms remit, many patients fail to achieve satisfactory social functioning or subjective recovery. This highlights a critical gap between symptomatic improvement and broader recovery outcomes [7].

In recent years, the concept of recovery in BD has expanded beyond affective symptom reduction to encompass functional, cognitive, and personal recovery and improved QOL [8,9]. Social and occupational dysfunctions are common in BD and strongly predict long-term outcomes [7,10]. Cognitive impairments affecting attention, executive function, and processing speed are frequently apparent during depressive episodes and may persist into euthymia, contributing to enduring disability [11,12]. Furthermore, the concept of personal recovery, defined as a subjective sense of hope, autonomy, and meaning, has gained increasing recognition as a key outcome in mood disorders [13]. However, the biological mechanisms underlying multidimensional recovery across these domains remain poorly understood.

Accumulating evidence suggests that immune-inflammatory dysregulation plays an important role in the pathophysiology of BD. Studies have identified increased levels of interleukin (IL)-6, tumor necrosis factor alpha (TNF-α), and IL-1β, and alterations in IL-2 signaling in BD, particularly during acute episodes [14,15,16,17]. These inflammatory changes appear to be more pronounced during the depressive phase and may affect neurobiological processes relevant to mood regulation and cognition [18,19].

Other neurotrophic and angiogenic factors that have been implicated in BD include brain-derived neurotrophic factor (BDNF) and vascular endothelial growth factor (VEGF). Reduced peripheral levels of BDNF have been reported during depressive episodes and are thought to reflect impaired neuroplasticity [20]. VEGF is a key regulator of angiogenesis and neurogenesis that has been associated with mood disorders. It is thought to influence brain function through interactions with inflammatory pathways [21,22]. Importantly, inflammation, BDNF, and VEGF form a biologically interconnected network that affects synaptic plasticity, cerebral perfusion, and neural resilience [22].

Despite these advances in our understanding of the role of neurotrophic and inflammatory factors in BD, biomarker studies have largely focused on their associations with the severity of affective symptoms. Few investigations have examined their relationships with functional outcomes, cognitive performance, personal recovery, or QOL in BD [23]. Even fewer have simultaneously assessed inflammatory cytokines alongside neurotrophic and angiogenic markers within a recovery-oriented framework. As a result, it is unclear whether these biomarkers are merely state-dependent correlates of depressive symptoms or broader biological substrates underlying impaired recovery.

Clarifying the relationships between inflammatory cytokines, BDNF, VEGF, and multidimensional recovery outcomes may provide critical insights into the mechanisms of recovery in BD. This information may also contribute to the development of biomarker-informed, recovery-oriented treatment strategies. The aim of this study is to conduct an exploratory analysis of the associations between inflammatory cytokines (IL-6, TNF-α, IL-2, and IL-1β), BDNF, and VEGF and clinical outcomes, including social functioning, cognitive functioning, personal recovery, QOL, and depressive symptom severity, in patients with BD.

## 2. Materials and Methods

### 2.1. Participants

This cross-sectional study enrolled patients with a clinical diagnosis of BD from Fukuoka University Hospital, Amagi Hospital, Abrayama Hospital, Fukuma Hospital, Shiranui Hospital, Mito Hospital Niji to Umino Hospital, Wakahisa Hospital, and Gannosu Hospital. We utilized baseline data from the CERF-BD trial [24]. Twenty-four Japanese patients with BD were enrolled. The inclusion criteria were as follows: (1) A diagnosis of BD based on the criteria of the Diagnostic and Statistical Manual of Mental Disorders, Fifth Edition (DSM-5); (2) A current depressive episode; (3) A score of 6–15 points on the Quick Inventory of Depressive Symptomatology-Self-Report (QIDS-SR); and (4) Aged between 20 and 65 years. The exclusion criteria were as follows: (1) Intellectual disability; (2) A history of cranial trauma with loss of consciousness; (3) A physical disease known to cause mental health problems; (4) A pervasive developmental disorder; (5) Pregnancy or breastfeeding; (6) Severe or uncontrolled cardiovascular risk factors, such as unstable coronary artery disease, uncontrolled hypertension, malignant ventricular arrhythmia, atrial fibrillation, exercise-induced ischemia, and ventricular failure; (7) Another significant medical condition, including, but not limited to, chronic or recurrent respiratory, gastrointestinal, neuromuscular, or musculoskeletal problems that interfere with exercise; and (8) An inflammatory disease.

### 2.2. Clinical Assessments

#### 2.2.1. Depressive Symptoms

The severity of each participant’s depressive symptoms was measured using the QIDS-SR [25], which consists of 16 questions. The total possible severity score ranges from 0 to 27. The higher the score, the more severe the depressive symptoms.

#### 2.2.2. Psychosocial Functioning

To evaluate psychosocial functioning, we administered the Functional Assessment Short Test (FAST) [26]. The FAST includes 24 items across six functional domains. These are autonomy (four items), occupational functioning (five items), cognitive functioning (five items), financial issues (two items), interpersonal relationships (six items), and leisure time (two items). For each item, the degree of difficulty experienced over the past 14 days is rated on a four-point scale: 0 (no difficulty), 1 (mild difficulty), 2 (moderate difficulty), or 3 (severe difficulty). Item scores are summed to yield the total FAST score, which ranges from 0 to 72 points, with higher scores indicating greater impairment.

#### 2.2.3. Cognitive Functioning

To assess cognitive impairment, we used the Thinc-it tool [27]. This is a digital cognitive assessment tool. The complete THINC-it test set includes Spotter, Symbol Check, Codebreaker, Trails, and the Depression Deficit Questionnaire-5 Items (PDQ-5-D).

Spotter: This test is used to assess attention and response time. We used the average reaction times on this test in our analysis. Longer reaction times on the Spotter task indicate poorer attentional performance.

Symbol Check: This test measures working memory (one-back task) and attention. We used the number of correct answers on this test in our analysis.

Codebreaker: This test is modeled on the digit symbol substitution test. It measures attention, perceptual speed, motor speed, visual scanning, and memory. We used the number of correct answers on this test in our analysis.

Trails: This test assesses executive function. It is based on the Trail-Making Test B. We used the total time taken by each participant on this test in our analysis. Longer completion times on the Trails task indicate poorer executive functioning.

The THINC-it tool also includes an additional five-item component taken from the Perceived Deficit Questionnaire (PDQ-5-D). Higher PDQ-5-D scores indicate greater subjective cognitive dysfunction.

#### 2.2.4. Personal Recovery

To assess personal recovery, we used the Japanese version of the Questionnaire about the Process of Recovery (QPR-J). The translated scale has been tested and shows good reliability and validity [28]. Higher scores indicate greater recovery.

#### 2.2.5. QOL

The World Health Organization-Quality of Life Assessment-26 (WHO-QOL-26) is the Japanese version of the WHO Quality of Life-Brief Version. The translated version has been tested and validated. The WHO-QOL-26 comprehensively measures subjective well-being and comprises 24 items across four domains: physical health, psychological health, social relationships, and environment. It also includes two items that assess overall QOL and general health. Each item is rated on a five-point Likert scale ranging from one (not at all/very dissatisfied) to five (a lot/very satisfied). Higher WHO-QOL26 scores indicate better quality of life.

### 2.3. Samples

Venous blood (7 mL) was drawn between 06:00 and 09:00 using Venoject^®^ II vacuum blood collection tubes (Terumo Corp., Tokyo, Japan) containing ethylenediaminetetraacetic acid disodium salt (EDTA-2Na). Plasma fractions were separated at 2500 g for 20 min in RT within 12 h. The plasma samples were stored at −80 °C until use. The quantification of plasma components (TNFα, IL-6, IL-1β, IL-2, BDNF, and VEGF-A) was outsourced to Eurofins GeneticLab Co., Ltd. (Sapporo, Japan) and performed in singlicate using an LXSAHM Human Magnetic Luminex Assay (R&D Systems, Minneapolis, MN, USA). The plasma samples were kept frozen during transport. The range of the standard curves for each component in the nominal specifications of the Luminex assay were TNFα: 8.23–2000 pg/mL, IL-6: 4.53–1100 pg/mL, IL-1β: 17.7–4300 pg/mL, IL-2: 30.9–7500 pg/mL, BDNF: 20.6–5000 pg/mL, VEGF-A: 11.5–2800 pg/mL, and the sensitivities were TNFα: 1.2 pg/mL, IL-6: 1.7 pg/mL, IL-1β: 0.8 pg/mL, IL-2: 1.8 pg/mL, BDNF: 0.320 pg/mL, VEGF-A: 0.99 pg/mL. Unmeasurable samples with extremely low concentrations were included in the analysis under the assumption of a 0 pg/mL concentration.

### 2.4. Statistics

JMP Student Edition, v. 18.2.2 (Cary, NC, USA), was used for statistical analysis. The normality of each variable’s distribution was evaluated using the Shapiro–Wilk test. Since nonparametric variables were included, we evaluated correlations between variables using Spearman’s rank correlation coefficient. Spearman’s correlation coefficients were calculated to assess associations between variables. Given the large number of comparisons, *p*-values were adjusted using the Benjamini–Hochberg false discovery rate (FDR) procedure. Given the exploratory nature and limited sample size of this pilot study, multivariable analyses were not performed to avoid model overfitting.

## 3. Results

### 3.1. Demographics

The clinical and sociodemographic characteristics of the participants are summarized in Table 1.

### 3.2. Associations Between Test Scores on Symptom Measures (Table 2)

We found significant correlations between subjective cognitive function and both depressive symptoms (QIDS-SR total score) (rho = 0.53, *p* = 0.0083) and WHO-QOL-26 total score (rho = −0.56, *p* = 0.0042). We also found a relationship between depressive symptoms and WHO-QOL-26 total score (rho = −0.43, *p* = 0.038). After FDR correction, the associations between FAST score and PDQ-5-D, Symbol check and Codebreaker, and Codebreaker and Trails remained statistically significant.

**Table 2:** Associations between psychometric test scores in patients with bipolar disorder (Spearman’s rho and FDR-adjusted q values).

	FAST	QIDS-SR	WHO-QOL26	QPR	Spotter	Symbol Check	Codebreaker	Trails	PDQ-5-D
**FAST**	-	0.36, 0.24	−0.38, 0.09	−0.24, 0.39	−0.17, 0.96	0.13, 0.90	−0.01, 0.96	0.21, 0.78	**0.41** **, 0.045**
**QIDS-SR**		-	−0.43, 0.36	−0.23, 0.78	−0.2, 0.39	0.08, 0.86	−0.20, 0.70	−0.28, 0.78	0.53, 0.09
**WHO-QOL26**			-	0.39, 0.23	−0.04, 0.96	0.10, 0.96	−0.03, 0.96	0.18, 0.96	−0.56, 0.05
**QPR**				-	−0.05, 0.96	−0.02, 0.94	−0.11, 0.85	0.23, 0.90	−0.22, 0.78
**Spotter**					-	−0.51, 0.23	−0.50, 0.06	0.47, 0.28	−0.04, 0.94
**Symbol check**						-	**0.** **7** **0, 0.002**	−0.37, 0.39	0.12, 0.85
**Codebreaker**							-	**−0.** **7** **0, 0.001**	0.05, 0.90
**Trails**								-	−0.14, 0.96
**PDQ-5-D**									-

Footnotes: For FAST, PDQ-5-D, Spotter, and Trails, higher scores indicate worse functioning or cognitive performance. For WHO-QOL26 and QPR, higher scores indicate better quality of life and recovery. Bold text indicates FDR-adjusted q values < 0.05.

### 3.3. Associations Between Psychometric Test Scores and Cytokine (TNF-Alpha, IL-6, IL-1β, IL-2), BDNF, and VEGF Levels

Table 3 shows the relationships between participants’ scores on each clinical symptom assessment and their cytokine (IL-2, IL-6, TNF-α, IL-1β), BDNF, and VEGF levels. There were positive correlations between plasma IL-6 levels and Trails scores (rho = 0.43, *p* = 0.0068). We found a positive correlation between plasma IL-1β levels and WHO-QOL26 scores (rho = 0.54, *p* = 0.02). We also found a negative correlation between plasma VEGF levels and Spotter scores (rho = −0.42, *p* = 0.042). After FDR correction, only the association between IL-1β and QOL remained statistically significant.

### 3.4. Comparison of BP I and BP II

We additionally explored potential differences between patients with bipolar I disorder and bipolar II disorder. No significant differences were observed in inflammatory biomarkers, cognitive measures, psychosocial functioning, or recovery-related outcomes between the two groups.

## 4. Discussion

In this exploratory pilot study, we investigated the associations between inflammatory cytokines, neurotrophic and angiogenic factors, and multidimensional clinical outcomes, including depressive symptoms, psychosocial functioning, cognitive performance, personal recovery, and QOL in patients with BD. Several important findings emerged. First, subjective cognitive dysfunction was significantly associated with depressive severity and poorer QOL. Second, among the examined biomarkers, plasma IL-6 levels were associated with poorer executive functioning, whereas VEGF levels were associated with attentional performance. However, after correction for multiple comparisons using the false discovery rate (FDR) procedure, only the association between IL-1β and QOL remained statistically significant. Taken together, these findings suggest that clinical outcomes in bipolar depression are closely interconnected, whereas biomarker associations should be interpreted cautiously and considered exploratory.

Consistent with the previous report, our findings further clarify the close relationships among depressive symptoms, subjective cognition, psychosocial functioning, and QOL in BD [5,7]. However, its association with broader psychosocial functioning was modest, suggesting that symptomatic remission does not necessarily translate into functional recovery in BD [7,29]. Patients frequently experience persistent impairments in the occupational and social domains during periods when depressive symptoms are mild, indicating that recovery is inherently multidimensional [29]. Our findings further support this by demonstrating that subjective cognitive dysfunction is more strongly related to depressive symptoms and QOL than scores on objective cognitive measures.

The association between higher IL-6 levels and poorer cognitive performance on the Trails task was particularly noteworthy. This finding is broadly consistent with previous evidence suggesting that inflammatory processes contribute to cognitive dysfunction in mood disorders [19,23,30]. IL-6 has been implicated in altered neuroplasticity, neurotransmitter metabolism, and the disruption of frontal-limbic and frontal-striatal circuits [19,31]. Similarly, the association between VEGF and attentional performance may reflect the role of VEGF in neurogenesis, angiogenesis, and neurovascular coupling. Previous preclinical and clinical studies have suggested that VEGF may support synaptic plasticity and cerebral perfusion, thereby contributing to cognitive performance [20,21]. However, these biomarker associations did not remain significant after FDR correction and therefore should be considered preliminary and hypothesis-generating.

Importantly, after FDR correction, only the positive association between IL-1β and WHO-QOL26 scores remained statistically significant. Although this finding appears counterintuitive given the commonly reported association between inflammation and poorer outcomes in mood disorders, increasing evidence suggests that inflammatory signaling in bipolar disorder may be heterogeneous, state-dependent, and not uniformly maladaptive [18,22]. Neuroimmune models have proposed that cytokine activation may, in some contexts, reflect compensatory or regulatory biological responses rather than simply illness severity [22]. In addition, subjective quality of life represents a multidimensional construct influenced not only by biological burden but also by resilience, adaptation, coping style, and illness perception. Therefore, peripheral inflammatory markers and subjective well-being may not necessarily demonstrate simple linear relationships.

Although exploratory subgroup analyses comparing bipolar I and bipolar II disorder were conducted, no significant differences were observed. However, the sample size within each subgroup was small, limiting statistical power to detect potential subtype-specific effects. Therefore, possible differences between bipolar subtypes should be examined in larger studies.

The present study had several limitations. First, this study is the relatively small sample size, which may have reduced statistical power to detect moderate associations. Therefore, correlations should be interpreted with caution. The findings should be considered exploratory and require replication in larger, well-powered studies. A second, important limitation of this study is the potential influence of psychotropic medications on inflammatory cytokines, BDNF, and VEGF levels. Mood stabilizers and antipsychotics have been reported to modulate immune-inflammatory and neurotrophic pathways, and medication effects could therefore have confounded the observed associations. Because of the relatively small sample size and heterogenous treatment regimens, we were unable to adequately control for medication effects in the present analyses. Accordingly, the findings should be interpreted cautiously and considered preliminary. Third, factors such as dietary habits and socioeconomic status that could affect cytokine, BDNF, and VEGF levels were not considered. Therefore, our findings should be considered preliminary in nature. However, as our participants were hospitalized patients, their recent dietary habits would have been reasonably consistent across the cohort. Fourth, the study was not designed to assess the potential influence of the various mood stabilizers and antipsychotics that the participants were receiving on the levels of the biological markers assessed. Previous studies have reported that some mood stabilizers and antipsychotics can affect the BDNF, VEGF, and cytokine networks [22,32]. However, we did not find the associations between the dose or duration of treatment with atypical antipsychotic drugs and mood stabilizers and biological marker levels that have been observed in more diverse populations. Moreover, several potential confounding factors, including psychotropic medications, BMI, metabolic status, illness duration, smoking, and physical activity, may have influenced biomarker levels in the present study. Because of the relatively small sample size, we were unable to perform adequately powered multivariable analyses to control for these factors. Therefore, the observed associations should be interpreted cautiously and considered exploratory in nature. Future studies with larger samples are needed to clarify the independent effects of these biomarkers after adjustment for potential confounders. Finally, BDNF, VEGF, and cytokines were only assayed at one time point in our sample.

## 5. Conclusions

The present study contributes to the growing literature examining recovery-related outcomes in bipolar depression. In particular, our findings highlight the strong interrelationships among depressive symptoms, subjective cognition, psychosocial functioning, and QOL. Although the biomarker findings remain preliminary, they suggest that inflammatory and neurovascular processes may contribute to cognitive and functional outcomes in bipolar depression and warrant further investigation in larger, longitudinal studies.

## Figures and Tables

**Table 1:** Clinical and demographic characteristics of the participants.

Variable	Mean ± SD (Range)
Age (year)	43.0 ± 13.0 (21–64)
Sex (m/f)	15/9
Number of hospitalizations	3.9 ± 3.1
Education (year)	13.3 ± 2.8
Age at onset	29.3 ± 11.1
Duration of illness	13.5 ± 9.4
BPI/BPII	10/14
BMI (Kg/m^2^)	27.3 ± 5.2
FAST total score	31.0 ± 9.5 (19–59)
QIDS-SR total score	11.1 ± 3.1 (6–15)
WHO-QOL total score	74.2 ± 13.3 (50–103)
QPR total score	66.3 ± 12.3 (44–92)
Spotter (ms)	728.3 ± 325.8
Symbol check	21.3 ± 11.3
Codebreaker	44.7 ± 18.9 7–75)
Trails (sec)	37.8 ± 22.9 (12.3–94.1)
PDQ-5-D	6.6 ± 3.4 (2–13)
TNF-alpha (pg/mL)	2.7 ± 1.3 (1.2–6.2)
IL-6 (pg/mL)	2.5 ± 1.3 (0.9–5.4)
IL-1 β (pg/mL)	4.3 ±3.4 (0–15.3)
IL-2 (pg/mL)	1.3 ± 2.5 (0–10.4)
BDNF (pg/mL)	6138.6 ± 5083.1 (357.1–22,498.3)
VEGF-A (pg/mL)	129.8 ± 185.9 (1.6–813.6)
Medication	n
Lithium	6
Valproic acid	7
Lamotrigine	6
Antipsychotics	18
Antidepressants	4

**Table 3:** Associations between psychometric test scores and biological marker levels in patients with bipolar disorder (Spearman’s rho and FDR-adjusted q values).

	TNF-α	IL-6	BDNF	IL-1β	IL-2	VEGF-A
**FAST**	−0.01, 0.97	0.41, 0.42	0.21, 0.85	−0.35, 0.64	−0.09, 0.96	0.29, 0.47
**QIDS-SR**	0.2, 0.94	0.001, 0.95	0.03, 0.94	−0.16, 0.96	0.26, 0.95	0.04, 0.96
**WHO-QOL26**	−0.04, 0.94	−0.31, 0.40	0.36, 0.39	**0.54** **, 0.006**	0.09, 0.93	−0.07, 0.96
**QPR**	0.14, 0.96	0.15, 0.86	−0.02, 0.85	0.15, 0.39	−0.07, 0.96	−0.16, 0.94
**Spotter**	−0.09, 0.96	0.35, 0.36	−0.07, 0.72	−0.22, 0.96	−0.03, 0.78	−0.42, 0.36
**Symbol check**	0.05, 0.96	−0.12,0.96	−0.17, 0.96	0.02, 0.96	−0.25, 0.85	−0.06, 0.96
**Codebreaker**	−0.09, 0.85	−0.15, 0.90	−0.3, 0.85	−0.03, 0.96	−0.08, 0.90	−0.26, 0.78
**Trails**	0.01, 0.78	0.43, 0.85	0.36, 0.36	0.01, 0.96	−0.03, 0.94	0.06, 0.38
**PDQ-5-D**	0.04, 0.96	−0.09, 0.97	−0.21, 0.96	−0.22, 0.78	−0.16, 0.96	0.1, 0.56

Footnotes: For FAST, PDQ-5-D, Spotter, and Trails, higher scores indicate worse functioning or cognitive performance. For WHO-QOL26 and QPR, higher scores indicate better quality of life and recovery. Bold text indicates FDR-adjusted q values < 0.05.

## Data Availability

The data are not publicly available due to privacy and ethical restrictions (i.e., we did not obtain informed consent on the public availability of raw data).

## Abbreviations

The following abbreviations are used in this manuscript:

BD	Bipolar disorder
BDNF	Brain-derived neurotrophic factor
FAST	Functional Assessment Short Test
PDQ	Perceived Deficit Questionnaire
QOL	Quality of life
QPR	Questionnaire about the Process of Recovery
VEGF	Vascular endothelial growth factor
